# Hospitals from the Patients' Point of View

**Published:** 1914-03-07

**Authors:** 


### HOSPITALS FROM THE PATIENTS' POINT OF VIEW.

(Contributions to this Section are Invited.)

### Experiences in a Provincial Isolation Hospital.

I HAD lived for years in "rooms," and long before my attack of scarlet fever had resolved never to be nursed, if illness came, by even the tenderest-hearted of landladies. There is neither the time nor the facility for nursing in the average boarding-house. So as soon as the first shock of surprise was over I begged my doctor to arrange for my accommodation at the local isolation hospital.

That was at ten o'clock on Monday morning, and about three o'clock on Tuesday afternoon I awoke to find a nurse in my room filling up a card with certain "instructions for the patient's friends" for my landlady. I was hurried into a dressing-gown, slid my feet into a pair of soft slippers, and tastefully robed in the hospital blanket brought by nurse, descended to the waiting ambulance brougham.

The first part of that drive I rather enjoyed; the febrile symptoms had mostly gone, and I felt fairly well. The experience was novel and amusing in its way, and it was a lovely warm sunny day in March.

I soon found there were other patients to bring; the first a young girl of fifteen or so from a school, the second a little girl from one of the city's slummiest streets, and the third another little girl from a mechanic's home, who was fully and daintily dressed to go away, even to a fresh blue ribbon in her hair. Only the humour of the situation struck me at the time, but when later on I learned the possibility and possible danger of cross-infection. I wondered whether the economy of this method was not secured at too great a risk.

By the time we reached the hospital the journey must have lengthened into pretty nearly six miles, and I was feeling faint and ill. However, soon after getting into bed (and after taking the routine "mag. sulph.") a nurse brought me a little bread and milk, which was very comforting. Then followed the routine hot bath, and I prepared to settle down for the night. Not, however, to sleep, I thought, for out of the twenty-four beds in the ward twenty were occupied by small boys, and the noise was like pandemonium. But they soon quieted down, and the gas was lowered, so sleep seemed nearer. That it did not come was partly due to the thinness of my pillow; to this day I cannot sleep with my head low, and neither that night nor during the whole of my stay in the hospital was an extra pillow to be had. There was one for every bed, and no more. Eventually I got more comfort by rolling up my dressing-gown as a supplementary bolster.

About 11.30 the house physician came round, and after a careful examination ordered me to be removed from the ward to the open corridor communicating with the next ward. To some people the change would no doubt have been a real hardship, but to a lover of the open air, like myself, the intense cold brought no discomfort, and the comparative quiet was a great relief. A long time afterwards I found that Doctor H—— had found certain spots on my skin of a "suspicious" type (seen in the dim light) and thought it safer for the other patients to send me outside.

Although the weather was very cold—so cold that one day the foot of my bed was powdered with snow—after the first day I felt little or nothing of it; there were plenty of blankets, and the nurses were most kind in providing a frequently renewed hot-water bottle.

In about a week chicken-pox broke out in the ward, and those of us who escaped infection were removed to another ward usually devoted to enteric and "mixed" cases. Here I had to go inside, and deeply regretted the change. The first two days the ward seemed intolerably stuffy, though, as a matter of fact, there was any amount of fresh air—too much for some of the patients.

A trace of albumin kept me in bed for ten days, nearly a fortnight in all; rather a long allowance for simple scarlet fever. Few of my fellow-patients had as long. Then came the day when I was allowed to get up, and quickly the day when it was possible to go out into the grounds.

Now I found the advantage of hospital life. At home I should have been secluded to one room at the top of the house in a narrow street, with little outlook, little or no exercise, and practically no society. Here I enjoyed all three, and if the society was that of men outside my own circle that was an advantage; I profoundly pity the man who cannot reconcile himself to that and so learn something of the mind of men who otherwise might remain strangers to him for ever.

I was in W—— Hospital for six weeks and a day, and was both glad and sorry to leave. There are a few suggestions I would like to make.

First as to the meals: The utensils were nearly all of enamelled iron—much of it badly chipped, and when this was so in the case of cups, they were quite unpleasant to use, and often a marked taste crept into the tea. Latterly I was favoured and had breakfast and tea-things of white china, but to be singled out was in itself unpleasant. I don't think any of the adult patients would use enamelled iron "crockery" in their own homes, and I felt, and still feel, they might well be trusted to preserve china things intact.

The food itself was very good indeed; as far as my observations went the quality was genuinely first class, and an attempt was made by the nurses to place it before those of us likely to appreciate it in an appetising way; I mean that the bread and butter was cut thin, and so on.

In kind, it was exceedingly monotonous. With regard to meat, that I do not complain of. It must be very difficult to provide meat for 150 patients outside the eternal beef and mutton. But the second course was always rice pudding (baked) and stewed prunes. I think the latter gave place to stewed rhubarb three times, but certainly not more. Stewed prunes, of course, are excellent food, but after the thirtieth day in succession one comes to look upon them with some want of enthusiasm.

There was no cake or jam provided for tea, and yet when one thinks how cheaply currant bread, or a plain type of dough cake, could be provided, surely it would be possible to supply it as a "treat," say, two or three times a week.

Supper was bread and milk, with as much sugar as fancy dictated, and as I was brought up (in a farmhouse) on that, I for one hailed it with joy. Several times broth was substituted, to the lively satisfaction of some of the patients, but for me it was a very late second-best.

It was always possible to have a cup of hot milk in the night if unable to sleep, and that often proved an extraordinary comfort. Some of the wards were such a distance from the kitchen that "hot" food arrived too cool to be really appetising.

Secondly, there was no kind of religious observance in any of the wards at any time. Sunday was monotonously like the other six days. No doubt most of the patients would welcome something of the kind, and I should think something might have been arranged. But I am sensible of the difficulty of exposing outsiders to infection. Thirdly, W—— Hospital is quite three miles from that part of the near-by town from which most of its patients come, and a long mile from the nearest tram—a mile in which two fairly steep hills have to be climbed—and some provision for restoring patients to their homes would be an immense boon. It cost me 4s. for a taxi, but of course very few of the patients could afford that.

For the rest, the attention from the nurses was extremely kind and, as far as I was competent to judge, fairly efficient; most of them were entirely new to nursing. The house physician was rather young, and no doubt would feel freer to unbend a little as he grew older.

In conclusion, the need for wards where patients can pay a part of their cost and enjoy some slight advantages is very great, and for a period of six to eight weeks £2 2s. a week (a sum I often see recommended) would be too much. A clerk or mechanic earning £2 or £2 10s. a week, with a wife and two or three children, ought not to have to hand over from twelve to sixteen guineas for an illness not requiring a surgeon or specialist.

